# “Our Choice” improves use of safer conception methods among HIV serodiscordant couples in Uganda: a cluster randomized controlled trial evaluating two implementation approaches

**DOI:** 10.1186/s13012-021-01109-z

**Published:** 2021-04-15

**Authors:** Glenn J. Wagner, Rhoda K. Wanyenze, Jolly Beyeza-Kashesya, Violet Gwokyalya, Emily Hurley, Deborah Mindry, Sarah Finocchario-Kessler, Mastula Nanfuka, Mahlet G. Tebeka, Uzaib Saya, Marika Booth, Bonnie Ghosh-Dastidar, Sebastian Linnemayr, Vincent S. Staggs, Kathy Goggin

**Affiliations:** 1 RAND Corporation, 1776 Main St., Santa Monica, CA 91105 USA; 2 School of Public Health, College of Health Sciences, Makerere University, Kampala, Uganda; 3 Department of Reproductive Medicine, Mulago Specialised Women and Neonatal Hospital, Kampala, Uganda; 4 Department of Disease Control and Environmental Health, Makerere University School of Public Health, Kampala, Uganda; 5 Children’s Mercy Research Institute, Children’s Mercy Kansas City, University of Missouri - Kansas City School of Medicine, 2401 Gillham Road, Kansas City, MO 64108 USA; 6 University of California Global Health Institute, Center for Women’s Health and Empowerment, 1234 Sunny Oaks Circle, Altadena, CA 91001 USA; 7 Department of Family Medicine & Community Health, University of Kansas Medical Center, Kansas City, KS 66160 USA; 8 The AIDS Support Organization, Kampala, Uganda; 9 Pardee RAND Graduate School, 1776 Main St., Santa Monica, CA 91105 USA

**Keywords:** HIV, Safer conception counseling, Contraception, Family planning, Uganda, Serodiscordant couples, Safer conception methods, Timed condomless intercourse, Manual self-insemination, Implementation approaches

## Abstract

**Background:**

Safer conception counseling (SCC) to promote the use of safer conception methods (SCM) is not yet part of routine family planning or HIV care. Guidelines for the use of SCM have been published, but to date there are no published controlled evaluations of SCC. Furthermore, it is unknown whether standard methods commonly used in resource constrained settings to integrate new services would be sufficient, or if enhanced training and supervision would result in a more efficacious approach to implementing SCC.

**Methods:**

In a hybrid, cluster randomized controlled trial, six HIV clinics were randomly assigned to implement the SCC intervention *Our Choice* using either a high (SCC1) or low intensity (SCC2) approach (differentiated by amount of training and supervision), or existing family planning services (usual care). Three hundred eighty-nine HIV clients considering childbearing with an HIV-negative partner enrolled. The primary outcome was self-reported use of appropriate reproductive method (SCM if trying to conceive; modern contraceptives if not) over 12 months or until pregnancy.

**Results:**

The combined intervention groups used appropriate reproductive methods more than usual care [20.8% vs. 6.9%; adjusted OR (95% CI)=10.63 (2.79, 40.49)], and SCC1 reported a higher rate than SCC2 [27.1% vs. 14.6%; OR (95% CI)=4.50 (1.44, 14.01)]. Among those trying to conceive, the intervention arms reported greater accurate use of SCM compared to usual care [24.1% vs. 0%; OR (95% CI)=91.84 (4.94, 1709.0)], and SCC1 performed better than SCC2 [34.6% vs. 11.5%; OR (95% CI)=6.43 (1.90, 21.73)]. The arms did not vary on modern contraception use among those not trying to conceive. A cost of $631 per person was estimated to obtain accurate use of SCM in SCC1, compared to $1014 in SCC2.

**Conclusions:**

More intensive provider training and more frequent supervision leads to greater adoption of complex SCM behaviors and is more cost-effective than the standard low intensity implementation approach.

**Trial registration:**

Clinicaltrials.gov, NCT03167879; date registered May 23, 2017.

**Supplementary Information:**

The online version contains supplementary material available at 10.1186/s13012-021-01109-z.

Contributions to the literature

- This hybrid cluster randomized controlled trial is the first to examine a multi-level safer conception counseling intervention for HIV serodiscordant couples implemented in the standard manner used in resource constrained settings vs. a higher intensity training and supervision approach.
- Findings demonstrated that the implementation approach with more provider training and more frequent supervision resulted in greater use of safer conception methods and was more cost-effective than the standard implementation approach.
- Regardless of implementation approach, the *Our Choice* intervention was more efficacious than usual care, demonstrating a large magnitude of effect.
- This is the first cost-effectiveness analysis of a multi-level safer conception counseling intervention and the first to include implementation strategy costs.
- Findings that adequate training and ongoing supervision are essential in promoting adoption of this complex health behavior and ultimately more cost-effective addresses gaps in the literature and may be applicable to the management of many other chronic illnesses.

## Background

Approximately 40% of HIV-infected women in Uganda become pregnant after HIV diagnosis [1, 2], and roughly half of these pregnancies are planned [2]. With 60% of HIV-affected couples in Uganda being serodiscordant [3], comprehensive family planning (FP) services are needed to help persons living with HIV (PLHIV) and their partners make informed childbearing decisions, and use effective methods for either safely conceiving or preventing unplanned pregnancies.

Despite FP services being integrated into HIV care, providers rarely discuss childbearing with clients prior to pregnancy [4]. HIV risk for serodiscordant couples is nearly eliminated by effective use of antiretroviral therapy (ART) [5], and while most are on ART [6], over a third of those on ART have unsuppressed viral load [6], and few seronegative partners in sub-Saharan Africa have access to pre-exposure prophylaxis (PrEP) [7, 8]. Safer conception methods (SCM) complement ART in promotion of safer conception, but knowledge of SCM is poor [9], and a prior study found that 35% of 400 PLHIV trying to conceive used SCM [10].

Providers could facilitate an informed childbearing decision-making process with periodic childbearing discussions. Clients could then be offered contraception to prevent pregnancy, or safer conception counseling (SCC) for effective SCM use if trying to conceive. SCC guidelines for PLHIV exist [11], but have not resulted in integration of SCC with either FP or HIV care. No randomized controlled trials of SCC were identified in a 2018 systematic review [12]; however, a prospective observational cohort study of 334 couples offered SCC found that many were able to use SCM to successfully conceive without any seroconversions [13]. It is also unknown whether the standard approach for integrating new services commonly used in resource constrained settings is sufficient to promote use of SCM, or whether more intensive training and supervision is needed, as suggested by studies of implementation approaches to increase complex health behaviors [14].

We conducted a hybrid cluster randomized controlled trial that compared usual care to two modes of implementing a SCC intervention named *Our Choice.* The study had two main objectives: (1) to determine the efficacy of *Our Choice* versus usual care on the primary outcome of accurate use of SCM or modern contraceptives consistent with client’s reproductive goal, and (2) to evaluate high versus low intensity approaches (differentiated by amount of training and frequency of supervision) to implementing *Our Choice* in terms of effects on the primary outcome and cost-effectiveness. We hypothesized that *Our Choice* would result in greater use of the appropriate reproductive method than usual care, and the more intensive implementation approach would lead to better uptake than the less intensive implementation approach, and be more cost-effective.

## Methods

### Study design

The study design was a three-arm cluster-randomized controlled trial conducted at six HIV clinics operated by The AIDS Support Organization (TASO) across 6 districts of Uganda (Wakiso, Masaka, Mbale, Jinja, Rukugiri, Mbarara). A cluster design was used to limit risks of contamination biases across treatment conditions.

#### Randomization and masking

Using a blind manual drawing, clinics were randomly assigned to one of three conditions by the project director: one of two implementation approaches for integrating SCC into FP services (more intensive=SCC1, less intensive=SCC2) or usual care (existing FP services). Allocation was neither concealed to providers nor individual participants (although clients did not know if their clinic was SCC1 or SCC2). The assigned condition was applied clinic-wide for all clients.

##### Power analysis

With a planned sample size of 400, enrolled evenly across 6 sites, and using an intra-class correlation (ICC) of 0.01 and assumed attrition of 10% attrition at month 12, our power analysis determined we would have > 80% power (2-tailed test) to detect a 4.5 percentage point difference (small effect size) for our comparison of the usual care arm to the combined SCC1 and SCC2 arms on the primary outcome, and a 7 to 8 percentage point difference between the SCC1 and SCC2 intervention arms.

Participants completed assessments at baseline, month 6, and month 12. If the client experienced a pregnancy by month 12, the final study assessment was conducted approximately 1 month after pregnancy completion. Participants were followed as long as they were still in a relationship with the partner they enrolled with at baseline; if they separated from this partner mid-study, the next assessment would be their final, unless they had an ongoing pregnancy which would lead to an assessment at the completion of that pregnancy. The protocol was approved by Institutional Review Boards at TASO and RAND, and described in a prior publication [15]. The research conformed to the principles embodied in the Declaration of Helsinki. The trial was registered with the NIH clinical trial registry (clinicaltrials.gov) and assigned the number NCT03167879.

#### Patient and public involvement

HIV clients provided input into the intervention development and outcome measures through participation in prior research that involved piloting the intervention and formative research including focus groups and in-depth interviews. Furthermore, HIV clients who volunteer as “expert clients” at the clinic sites were involved in aspects of implementing the intervention including community outreach and childbearing screening. These same clients will also be involved in dissemination of the study findings.

### Study setting and participants

TASO is the oldest indigenous non-governmental organization (NGO) in Uganda providing comprehensive HIV care. Each site provides care to 6,000-8,000 clients and has a staff of 15-20 medical providers. Clients were eligible if they met the following criteria: (1) in a serodiscordant relationship (partner’s HIV-negative status confirmed by rapid HIV test prior to enrollment), (2) of reproductive age (men age 15-60 years; women age 15-45), (3) considering childbearing with their partner (determined via triage screening item), (4) not currently pregnant (determined by a pregnancy test prior to enrollment), and (5) reports having disclosed HIV status to partner. Recruitment was stratified by sex to ensure a 50/50 balance in the overall study enrollment (not by study arm), and took place between July 2017 and January 2019. Clients who were potentially eligible were informed of the study by clinic staff and referred to the study coordinator for consent procedures. All enrolled participants provided written informed consent.

### Intervention conditions

#### Our Choice

Informed by our earlier research [10, 16] and guided by an ecological adaptation of the Information, Motivation and Behavioral skills (eIMB) model of behavior change [17], we developed a multi-component, structured intervention that engages HIV clients and their partners with fertility desires in SCC. The goal of the *Our Choice* intervention is for providers to facilitate an informed childbearing decision-making process and support each couple’s decision with training on the use of contraception or SCM in accordance with their reproductive goal. The counseling not only provides guidance and support for accurate use of SCM but also helps ensure that couples who opt not to seek pregnancy receive support to use a modern contraceptive rather than relying solely on condoms which are often inconsistently used. The intervention components are as follows: (1) client outreach to increase awareness and uptake of services, (2) routine screening of childbearing desires at triage, and (3) provision of SCC, starting with an initial consult conducted by an HIV counselor to facilitate an informed decision from the couple to pursue or delay pregnancy, followed by subsequent referral to FP nurses for either provision of contraception or monthly SCC sessions (see Supplement Figure 1). The client’s partner was encouraged to attend sessions, if possible. SCC was implemented by trained FP nurses, using a structured protocol and manual, the content of which is summarized in Supplement Table 1.

#### Low vs. high intensity approaches to implementation

*Our Choice* was implemented using two approaches, SCC1 and SCC2; both used the same content, manual and tools, but differed on the method, duration, and frequency of training and supervision of FP nurses and HIV counselors (see Supplement Table 2). SCC1 was the more intensive approach, with the study team providing initial training of providers over 2 days and supervision starting twice-a-month before transitioning to monthly contact at month 6. SCC2 was less intensive and followed the standard model used by the Uganda Ministry of Health (MoH) to integrate new services: initial 1 day training and quarterly supervision sessions (although in reality supervision occurred every 6-9 months) provided by MoH supervisors who had been trained by the study team.

#### Usual care control

Control sites received no training and provided FP services as usual, with no use of routine screening of childbearing desires or SCC.

### Measures

Assessments included measures of the primary outcome (see below), secondary outcomes (any use of SCM/contraception, pregnancy, partner seroconversion), and sociodemographic, HIV disease, and relationship/partner characteristics, as well as reproductive history and behaviors (see Table 1). Self-report measures underwent a translation, back-translation, and group consensus review process in Luganda and Runyakitara (local languages used in the study settings) [18], and were interviewer-administered using computer-assisted software. All measures were assessed at each assessment time point.

**Table 1:** Study measures

**Primary outcomes**	
Use of appropriate reproductive method to achieve stated reproductive goal (among whole sample)	Client’s self-reported use of accurate SCM (if trying to conceive) or modern contraceptives (if not trying to conceive), as defined below.
Accurate use of safer conception methods (among those trying to conceive)	Interviewer-rated criterion-based assessment of client’s self-reported use of timed condomless intercourse (TCI), manual self-insemination (MSI), or sperm washing.
Use of modern contraceptives (among those not trying to conceive)	Client’s self-reported use of modern contraceptives (birth control pills, medroxyprogesterone acetate injection, intrauterine device, implant, or tubal ligation/vasectomy).
**Secondary outcomes**	
Any use of SCM (among those trying to conceive)	Client’s self-reported use of the following: **TCI**: Did you have condomless or “live” sex only on the 3 days each month in which you/your partner were/was most fertile? **Sperm washing**: Did you/your partner pay for technology that cleanses your/your partner’s sperm or semen of the HIV virus? **MSI** (among female clients)**:** Did you/your partner ejaculate into a condom or container and then manually inject the semen into your/partner’s vagina?
Use of any method to prevent pregnancy (among those not trying to conceive)	Use of modern contraceptives, consistent condom use, or abstinence.
Pregnancy status	Whether or not female partner become pregnant during the study, confirmed via pregnancy test conducted by FP nurse.
Partner seroconversion	Partner HIV status based on rapid HIV test conducted by clinic staff at month 12 or at post-pregnancy assessment.
**Covariates**	
Demographics	Age, sex, and education level (whether or not any secondary education was completed) as self-reported by client.
HIV medical and care characteristics	HIV diagnosis date, ART status, most recent CD4 count and HIV viral load were abstracted from the clients’ clinic chart.
Relationship/partner characteristics	Marital status, length of relationship, co-habitation with partner, partner’s age, and whether partner was using HIV pre-exposure prophylaxis (PrEP), all assessed via client self-report.
Reproductive health history and behaviors	History of respondent or partner having biological children, having a child together, having been tested for infertility, or a health care provider ever telling them they may have fertility problems, and whether either had been diagnosed or (and treated for) a sexually transmitted infection in the prior 6 months, via client self-report.

### Primary outcome

The primary outcome was the accurate use of SCM [timed condomless intercourse (TCI), manual self-insemination (MSI), sperm washing] or contraception consistent with the client’s reproductive goal. This goal was determined at follow-up assessments by asking whether they had tried to conceive a child with their partner at any time in the past 6 months. Clients with missing responses were classified as trying to conceive if they endorsed “currently trying to conceive” at their most recent prior assessment. To assess accurate use of SCM, clients were asked in an open-ended format to describe exactly how they implemented their chosen SCM. Interviewers listened, probed for specific criterion not spontaneously mentioned, and rated the presence or absence of pre-defined criterion for each SCM (see Supplement Table 3). All criteria needed to be present for clients to be classified as accurately using the method.

To assess contraception use, clients were asked if they or their partner were currently using modern contraceptives, condoms, or abstinence to prevent pregnancy. Male participants were asked to consent to the interviewer calling their partner during the interview to assess use of contraception (calls were made in private and responses were not shared with the male partner). Accurate use of contraception was defined as using a modern contraceptive (i.e., birth control pills, medroxyprogesterone acetate injection, intrauterine device, implant, tubal ligation/vasectomy); condoms were not considered to be a modern contraceptive. This definition is consistent with FP practice in Uganda [19], as condom use is typically not consistent and therefore not reliable for preventing pregnancy, and it also requires cooperation from the male partner. We also assessed an alternative or secondary measure of contraception, such that participants who reported use of either modern contraception, consistent condom use, or abstinence were classified as using contraception.

### Data analysis

We conducted initial bivariate analyses to compare the study arms on baseline measures of sociodemographic characteristics, HIV disease characteristics, partner/relationship characteristics, and reproductive health history/behavior for participants. We assessed differences using chi-square or Fisher’s exact test (FET) for categorical variables, and two-tailed independent *t* tests for continuous variables. We repeated this analysis to compare those who completed the study with those who dropped out.

The primary analyses followed an intent-to-treat (ITT) approach (i.e., outcomes with missing data are designated as not engaging in the desired behavior or achieving the desired pregnancy status) and had two main objectives. First, to examine efficacy of *Our Choice*, we examined whether the primary outcome was more prevalent in the intervention group (SCC1 and SCC2 combined) compared to the usual care group. Second, we examined the relative efficacy of the two implementation approaches to administering *Our Choice* by comparing the effects of the higher intensity SCC1 versus the lower intensity SCC2. In both analyses, we first conducted bivariate chi-square or Fisher’s exact tests to assess associations between the outcome and treatment condition. We further tested for association using covariate-adjusted firth logistic regressions [20, 21]. Each logistic regression was adjusted for these covariates: age, sex, any secondary education, time since HIV diagnosis, marital status, length of relationship with partner, and whether the participant had a child with their partner. The model included fixed effects for site to account for clustering and allow for time-invariant differences across sites (a mixed model approach was not possible for outcomes with quasi-separation or all zero values as was observed in the usual care arm). We relied on the effect sizes (estimated by the differences across study arms) as demonstration of practically meaningful results, although statistical significance and *p* values were calculated and are presented. Effect sizes were estimated using Cohen’s *d*. This same approach was used to examine effects on the secondary outcomes. We replicated all analyses with study completers only. The results of this analysis (not presented) were very similar to that of the ITT analysis.

#### Cost-effectiveness analysis

We conducted a cost-effectiveness analysis (including implementation strategy costs [22];) based on program costs for personnel, supervision, training, and materials across both active study arms. Personnel costs included those for counselors, nurses, and expert clients, who over the course of the study were asked to recall how much time they spent weekly delivering intervention activities. Supervision costs were estimated by multiplying the duration of supervision sessions by the respective supervisor’s hourly salary and number of supervisory sessions conducted and including any transportation costs. Training costs included those for the initial trainings for providers (including transport and per diem), as well as any refresher trainings conducted, and costs incurred for training supervisors. Material costs included those for brochures, videos, phone top-ups for nurses, and transport for participants.

To examine the cost-effectiveness of the two different implementation approaches used in SCC1 and SCC2, we compared total intervention costs across the two active study arms for the primary outcome. Because this is one of the first analysis of SCC in this setting, we also assessed the costs for accurate use of SCM on its own among those trying to conceive. The cost-effectiveness of contraception provision in Uganda has already been established [23]. Because supervisors in SCC1 were research staff with significantly higher salaries than the MoH supervisors in SCC2, we conducted an additional more realistic “scale-up scenario” showing the costs of SCC1 if its supervisors’ salaries were the same as the MoH supervisors’ salaries. The cost-effectiveness ratio was calculated as the cost per participant divided by the relative effect size in that group compared to the usual care control. Costs in local currency were converted at 3600 Ugandan Shillings=US $1 and reported in 2020 prices adjusted for inflation.

## Results

### Sample characteristics

**Fig. 1:**
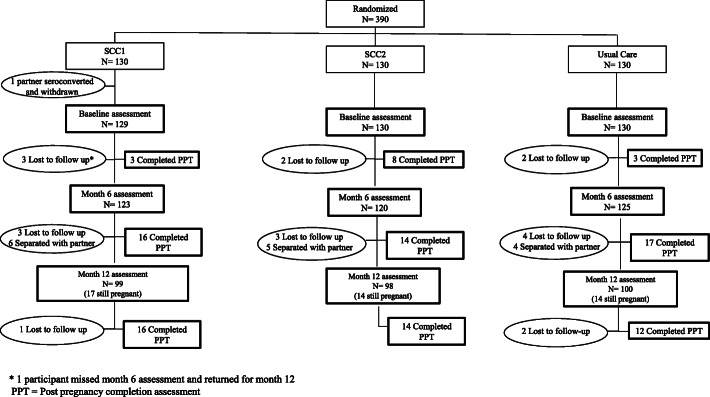
Consort diagram of study participants. Asterisk denotes 1 participant missed month 6 assessment and returned for month 12. PPT, post pregnancy completion assessment

In each of the three study arms, 130 clients consented and enrolled; the partner of one client in the SCC1 arm tested seropositive during screening, so this client was administratively removed. Therefore, a total of 389 clients comprise the study sample (129 in SCC1 and 130 in each of SCC2 and usual care arms). Figure 1 depicts the flow of participants through the assessment protocol. Sixteen participants (4.1%) were lost to follow-up either before the month 6 (*n*=6) or month 12 (*n*=10) assessments. Those lost to follow-up were similar to study completers with respect to baseline characteristics, except participants in SCC1 were more likely to be female compared to the usual care group (see Table 2). No harms or unintended negative events attributed to the intervention were reported, although 16 participants reported separating from their partner.

**Table 2:** Sample characteristics at baseline, by study completion and study arm

	Total sample (***n***=389)	Study completers (***n***=373)	Lost to follow-up (***n***=16)	***P*** value	SCC1 (***n***=129)	SCC2 (***n***=130)	Usual care (***n***=130)	***P*** value
	Mean (SD)/***n*** (%)	Mean (SD)/***n*** (%)	Mean (SD)/***n*** (%)		Mean (SD)/***n*** (%)	Mean (SD)/***n*** (%)	Mean (SD)/***n*** (%)	
**Sociodemographic characteristics**								
Age (years)	35.9 (8.2)	35.9 (8.1)	34.1 (10.5)	0.372	35.1 (7.2)	35.3 (8.0)	37.1 (9.1)	0.095
Female sex	195 (50.3%)	187 (50.1%)	8 (50.0%)	0.992	78 (60.5%)	66 (50.8%)	51 (39.2%)	**0.003**
Some secondary education	132 (33.9%)	126 (33.8%)	6 (37.5%)	0.758	34 (26.4%)	49 (37.7%)	49 (37.7%)	0.084
**HIV disease characteristics**								
Time since HIV diagnosis (years)	10.7 (8.7)	10.6 (8.7)	13.2 (9.7)	0.241	11.0 (9.1)	9.6 (8.6)	11.5 (8.5)	0.191
CD4 count (cells/mm^3^)^a^	518 (293)	524 (294)	391 (247)	0.142	537 (287)	535 (319)	483 (273)	0.400
Undetectable HIV viral load (*n*=315)^b^	265 (83.9%)	255 (84.2%)	10 (76.9%)	0.487	90 (87.4%)	78 (78.8%)	97 (85.1%)	0.229
Currently on ART	385 (99.2%)	369 (99.2%)	16 (100%)	0.718	129 (100%)	128 (98.5%)	128 (99.2%)	0.368
Time on ART (years)^c^	7.8 (7.1)	7.8 (7.1)	8·5 (7.2)	0.710	7.7 (7.5)	7.4 (7.0)	8.5 (6.7)	0.431
**Partner and relationship characteristics**								
Married to partner	326 (83.8%)	312 (83.7%)	14 (87.5%)	0.682	113 (87.6%)	102 (78.5%)	111 (85.4%)	0.114
Length of relationship (years)	9.9 (10.7)	10.0 (10.8)	9.6 (9.5)	0.907	9.3 (9.6)	9.4 (11.1)	11.0 (11.3)	0.363
Currently living with partner	344 (88.4%)	329 (88.2%)	15 (93.8%)	0.497	116 (89.9%)	114 (87.7%)	114 (87.7%)	0.811
**Reproductive history and behavior**								
Using modern contraceptives	71 (18.3%)	69 (18.5%)	2 (12.5%)	0.543	23 (17.8%)	27 (20.8%)	21 (16.2%)	0.622
Participant has biological children	349 (89.7%)	335 (89.8%)	14 (87.5%)	0.766	118 (91.5%)	112 (86.2%)	119 (91.5%)	0.261
Has had a child with partner	195 (50.1%)	187 (50.1%)	8 (50.0%)	0.992	59 (45.7%)	63 (48.5%)	73 (56.2%)	0.220

*SD* standard deviation, *PrEP* pre-exposure prophylaxis, *SCC1* safer conception counseling (high intensity approach), *SCC2* safer conception counseling (low intensity approach)

^a^A total of 252 participants had CD4 data available at baseline

^b^A total of 315 participants had viral load data available at baseline

^c^Among those on ART at baseline

#### Fidelity to *Our Choice* intervention

More participants in SCC1 received the initial safer conception consult [127 (98.4%) vs. 115 (88.5%); FET = .002] and follow-on SCC or FP services consistent with their reproductive goals [116 (89.9%) vs. 94 (72.3%); *p*=.001)], compared to SCC2. Among those who decided to pursue childbearing after the initial consult (*n*=105 in SCC1 and 87 in SCC2), 85.7% (*n*=90) of those in SCC1 received additional SCC sessions [mean (SD) of 3.4 (2.2) added sessions; median = 3; range 1-9], compared to 64.4% (*n*=56; *p*=.001) of those in SCC2 [mean (SD) of 3.0 (1.5) added sessions; median=3; range 1-7]. In SCC1, 91.3% (116/127) of the partners attended the initial safer conception consult, and 70.0% (63/90) attended at least one of the successive SCC sessions, while in SCC2, 60.9% (70/115) of partners attended the initial consult and 58.6% (51/87) attended at least one SCC session. Of those in SCC1 who received SCC sessions to promote childbearing, 18.8% (15 of the 80 who provided such data) never established a stable menstrual pattern to allow for the use of SCM, and another 30.0% (24/80) were only able to establish two stable menstrual cycles which allowed for only one SCM attempt. Comparable data were not consistently recorded in SCC2.

### Intervention effects on primary and secondary outcomes

Table 3 lists the group comparisons on all primary and secondary outcomes, with and without covariate adjustment, and using an ITT analysis (*p* values listed in the text are from the adjusted models).

**Table 3:** Intention-to-treat comparisons of the primary and secondary outcomes between the combined intervention group and usual care control, and between the two intervention groups (SCC1 vs. SCC2), with and without covariate adjustment

Overall	SCC1	SCC2	Sig. test(unadjusted)	Intervention effect(covariate adjusted)	Intervention(SCC1/SCC2)	Usual care control	Sig. test(unadjusted)	Intervention effect(covariate adjusted)
	***n*** (%)	***n*** (%)	Chi-sq	OR (95% CI)	***n*** (%)	***n*** (%)	Chi-sq	OR (95% CI)
**Among all participants in the sample (*n*=389)**								
***n***	**129**	**130**			**259**	**130**		
**Primary outcome**: Used SCM accurately if tryingto conceive or modern contraception if not tryingto conceive	35 (27.1%)	19 (14.6%)	0.013	4.50** (1.44-14.01)	54 (20.8%)	9 (6.9%)	<0.001	10.63** (2.79-40.49)
**Secondary outcomes**: Used SCM accurately iftrying to conceive or any contraception(incl condoms) if not trying to conceive	46 (35.7%)	33 (25.4%)	0.073	3.44* (1.31-9.02)	79 (30.5%)	27 (20.8%)	0.042	3.63** (1.38-9.61)
Partner seroconversion to HIV positive at studyendpoint	0 (0%)	1 (0.8%)	0.320	0.94 (0.01-75.66)	1 (0.4%)	0 (0%)	0.46	2.23 (0.02-257.6)
**Among those trying to conceive throughout study (*n*=212) or during one 6-month period (*n*=64): total** ***n*=276**								
***n***	**104**	**87**			**191**	**85**		
**Primary outcome**: Used TCI or MSI accurately	36 (34.6%)	10 (11.5%)	<0.001	6.43** (1.90-21.73)	46 (24.1%)	0 (0%)	<0.001	91.84** (4.94-1709)
**Secondary outcomes**								
Used any (un-named) strategy to reduce risk inconceiving	81 (77.9%)	61 (70.1%)	0.221	7.05** (2.07-23.99)	142 (74.3%)	26 (30.6%)	<0.001	27.17** (7.84-94.15)
Reported using SCM (TCI/MSI)	75 (72.1%)	50 (57.5%)	0.034	4.75** (1.64-13.71)	125 (65.4%)	4 (4.7%)	<0.001	494.4** (26.06-9378)
Reported using TCI	67 (64.4%)	42 (48.3%)	0.025	5.12** (1.86-14.50)	109 (57.1%)	4 (4.7%)	<0.001	424.6** (22.63-7968)
Reported accurate TCI	30 (28.8%)	9 (10.3%)	0.002	10.33** (2.60-41.08)	39 (20.4%)	0 (0%)	<0.001	121.9** (6.30-2360)
Reported using MSI (among females)	14/69 (20.3%)	8/39 (20.5%)	0.978	1.06 (0.27-4.11)	22/108 (20.4%)	0/34 (0%)	0.002	9.88 (0.48-201.9)
Reported accurate MSI	8/69 (11.6%)	1/39 (2.6%)	0.152	1.13 (0.13-9.54)	9/108 (8.3%)	0/34 (0%)	0.114	2.77 (0.11-67.26)
Became pregnant	32 (30.8%)	29 (33.3%)	0.760	0.44 (0.15-1.25)	61 (31.9%)	27 (31.8%)	0.902	0.73 (0.25-2.09)
**Among those not trying to conceive throughout study (*n*=113) or in one 6-month time period (*n*=64): total** ***n*=177**								
***n***	**48**	**61**			**109**	**68**		
**Primary outcome**: Using modern contraceptive	7 (14.6%)	12 (19.4%)	0.487	3.72 (0.37-37.48)	19 (17.4%)	12 (17.6%)	0.971	1.20 (0.22-6.501)
**Secondary outcomes**:								
Using modern contraceptive or always usecondoms or not having sex	28 (58.3%)	35 (56.5%)	0.92	1.07 (0.28-4.19)	63 (57.8%)	37 (54.4%)	0.658	0.52 (0.16-1.75)
Always use condoms	13 (27.1%)	16 (25.8%)	0.92	2.78 (0.50-15.63)	29 (26.6%)	18 (26.5%)	0.984	2.66 (0.57-12.44)
Did not become pregnant	44 (91.7%)	54 (88.5%)	0.589	1.40 (0.17-11.58)	98 (89.9%)	61 (89.7%)	0.965	0.18 (0.01-4.30)

Adjusted models controlled for site, age, sex, education, time since HIV diagnosis, marital status, length of relationship, and whether had child with partner

*SCM* safer conception method, *TCI* timed condomless sex, *MSI* manual self-insemination, *SCC1* safer conception counseling implemented with high intensity approach, *SCC2* safer conception counseling implemented with low intensity approach, *OR* odds ratio, *CI* confidence interval

**p<0.01

*p<0.05

#### Intervention effect on use of appropriate reproductive method

The combined intervention group (SCC1/SCC2) reported a higher rate of accurate SCM use (TCI or MSI; sperm washing was not reported by anyone) or modern contraception consistent with their reproductive goal, compared to the usual care group (20.8% vs. 6.9%; *p*=.001; Cohen’s *d*=.38). A similar result (30.5% vs. 20.8%; *p*=.04) was found when using the alternative definition of appropriate contraception (i.e., use of modern contraception, consistent condom use, or sexual abstinence; see Table 3).

#### Intervention effects on SCM use among those trying to conceive

Among the subgroup of 276 participants who reported trying to conceive throughout the study (*n*=212) or during one 6-month period (*n*=64), the combined intervention group reported higher accurate SCM use (24.1%) compared to the usual care group which had no accurate use of SCM (*p*=.002; Cohen’s *d*=.67). The combined intervention group also reported higher SCM use in general (regardless of accuracy), as well as higher TCI use, accurate TCI use, and MSI use (see Table 3). PrEP, which became available at three of the six sites midway through the final year of intervention implementation, was used by just 13 participants.

Among those in the intervention groups who reported using TCI or MSI, all received calls from the nurse counselor to inform them when the woman’s most fertile 3-day period was beginning; therefore, the high levels of inaccuracy for TCI were related to not knowing that the most fertile period was 3 days (30-43% across both follow-up assessments), not having condomless sex during the most fertile period (66-76%), and not always using condoms outside the most fertile period (67-74%). The inaccuracy of MSI use was mostly due to not knowing that the most fertile period was 3 days (38%), not injected semen into woman’s vagina during her most fertile period (69%), the woman not remaining in proper position for at least 30 min after the injection of semen (54%), and not always using condoms during sex (46%).

#### Intervention effects on modern contraception use among those trying to avoid pregnancy

In the subgroup of 177 participants who reported trying to avoid pregnancy throughout the study (*n*=113) or during one 6-month period (*n*=64), the combined intervention group (17.4%) demonstrated similar level of use of modern contraceptives as the usual care group (17.6%; *p*=.832). There were also no noteworthy differences when using the alternative definition of modern contraception, consistent condom use, or abstinence (see Table 3).

#### Intervention effects on pregnancy and partner seroconversion

Among the subgroup of 276 participants who tried to conceive, the pregnancy rate did not differ between those in the combined intervention group (31.9%) compared to the usual care group (31.8%; *p*=.902). We examined bivariate correlates of pregnancy during the study among the 265 study completers who reported trying to conceive at either of the follow-up assessments (see Supplement Table 4). Younger woman’s age was positively associated with having a pregnancy, while either partner having ever been tested for infertility or being told by a healthcare provider that they may have infertility problems was associated with not having a pregnancy. Among those who tried to avoid pregnancy, the proportion who did not get pregnant did not differ between the combined intervention group (89.9%) and usual care group (89.7%; *p*=.289).

Only one partner (in SCC2) tested HIV-positive at study endpoint. This couple was trying to conceive but only attended the initial safer conception consultation. The partner tested positive just before the month 6 follow-up, at which time the participant reported that his partner was also pregnant and that they had not been using SCM. The participant, who had been on ART for over 8 years and was virally suppressed when last tested (3 months prior to enrollment), reported condomless sex with his partner at both baseline and month 6.

### Effects of high (SCC1) versus low (SCC2) intensity intervention on primary and secondary outcomes

As shown in Table 3, SCC1 reported a higher rate of accurate SCM or modern contraceptive use (27.1%) consistent with their reproductive goal, compared to SCC2 (14.6%; *p*=.010; Cohen’s *d*=.31). Among those trying to conceive during the study, SCC1 had a higher rate of accurate SCM use (34.6% vs. 11.5%; *p*=.003; Cohen’s *d*=.56), as well as higher SCM use in general, accurate TCI use and TCI use, compared to SCC2; SCC1 had a similar pregnancy rate as that of SCC2 in this subgroup (30.8% vs. 33.3%; *p*=.760). SCC1 and SCC2 did not differ on contraception use or pregnancy rate, among those trying to avoid pregnancy.

### Cost-effectiveness of SCC1 vs. SCC2

The cost per client for use of appropriate reproductive methods (SCM or modern contraception, depending on reproductive goal) ranged from $105-176 across the “scale-up” and “actual” scenarios in SCC1, and was $78 in SCC2. The cost per client for accurate SCM use ranged from $130-218 across these scenarios in SCC1 and was $117 in SCC2. The cost-effectiveness ratio (or the cost per additional person treated to achieve the outcome) for use of appropriate reproductive method was $520-871 in SCC1 for the “scale-up” and “actual” costs scenarios, and $1014 in SCC2. For the accurate SCM outcome alone, it was $377-631 in SCC1 and $1014 in SCC2.

## Discussion

In what may be the first randomized controlled trial of a SCC intervention for PLHIV, *Our Choice* recipients self-reported greater use of recommended methods for achieving their reproductive goal, namely, accurate use of SCM or modern contraceptives, compared to participants who received usual care. Compared to usual care, *Our Choice* was most successful in enabling more clients to accurately use SCM when trying to conceive, but produced similar rates of self-reported modern contraception use among those not trying to conceive. Furthermore, the approach to implementing *Our Choice* that included more intensive training and supervision was more successful in increasing self-reported accurate SCM use than the standard implementation approach.

One of the main study objectives was to determine whether *Our Choice* was more efficacious than usual care in helping clients and their partners to accurately use SCM if they were trying to have a child. For such clients, the intervention’s magnitude of effect was large. Almost no participants in the usual care control group reported using SCM, which is not surprising given that SCC has not been integrated into standard FP practices, and thus awareness is poor [10, 16]. Nearly two-thirds of intervention participants reported using SCM, with most couples using TCI. Some also used MSI, which is noteworthy given that our qualitative research found that couples often resisted this method and were skeptical of its “unnatural” method of conception [10]. Just over one-third of those using SCM used the methods accurately, but more than half of these participants (or their partners) who used SCM exhibited irregular menstrual cycles that precluded attempting these methods and may indicate infertility [24, 25]. Future studies that include women with regular menstrual cycles are needed to establish better estimates of the true impact of SCC on accurate SCM use. Additionally, studies that examine the rate and any mutable causes of infertility would help to clarify the clinical value of widespread dissemination of SCC.

The other main objective of the study was to determine if the more intensive approach was needed to achieve better results. Our data clearly show that the high intensity approach (SCC1) resulted in greater reported use of SCM (TCI in particular). One third of SCC1 participants trying to conceive reported accurate SCM use, compared to just one tenth of those in SCC2. These findings may be attributed to the more extensive training (2 versus 1 day, and inclusion of motivational interviewing techniques) and once- or twice-monthly supervision in SCC1 compared to every 6-9 months in SCC2. Our process data also revealed the greater likelihood of SCC1 participants receiving the initial safer conception consult and follow-on SCC/FP services, as well as greater partner attendance in sessions, all of which may be attributable to the enhanced training and supervision. These findings contribute to evidence from implementation science that suggests the importance of adequate training and ongoing supervision to successfully integrate new services into routine care [14], as well as the body of literature suggesting that multi-level, properly implemented interventions are required to produce change in complex behaviors [26–28].

While the lower intensity SCC2 implementation approach has lower up-front costs and may therefore appear more sustainable, our findings indicate that the resources needed to implement the more intensive SCC1 approach were in fact more cost-effective in both the “actual” and “scale-up” scenarios. These findings further support the merits of implementation approaches that ensure adequate training and ongoing supervision support to providers when attempting to implement a new service, especially one that involves a complex health behavior like safer conception. We conducted the “scale-up” scenario to provide a more accurate estimate of what supervision would cost in a real-world application of the *Our Choice* program. SCC1 superviors garnered higher salaries because they were members of the research team who held advanced degrees. They received training to use standardized procedures and materials and followed the study protocol for providing technical assistance and ongoing support to providers, none of which required their advanced degrees or research training. Nevertheless, future studies should evaluate factors that predict optimal supervision. Policy evaluations for whether to integrate SCC into FP services for PLHIV will likely hinge on the important factors of ART use, viral suppression, and access to fertility testing. Unlike our study sample, scenarios where ART is not being used or HIV is not fully suppressed, and both partners are fertile, could greatly increase the value of implementing SCC to promote prevention of horizontal transmission and safe conception.

*Our Choice* did not have any effect on reported use of contraception among those trying to avoid pregnancy. The intervention was designed to promote use of modern contraception by helping couples reach a joint informed decision to not seek pregnancy, together with a referral to the FP nurse for usual care contraception services. The lack of an effect suggests that there was no added effect of the intervention beyond usual care, and the need for added components that specifically target contraception use. The overall reported use of modern contraception among those trying to avoid pregnancy was very low (15-20%) compared to the 68% found in a recent large study of HIV-positive Ugandan women seeking to prevent pregnancy [29]. However, our sample is distinct from the sample in that study and the general HIV population as our enrollment criteria required participants to report consideration of childbearing with their partner. While the couple may not yet have been in a position to pursue pregnancy, some desire to have a child was likely present, and may have served as a barrier to contraception use, particularly in the larger context of cultural pressure to have children [1, 30].

With nearly all participants being on ART, and only one partner seroconversion taking place during the study, we were not able to adequately assess the benefits of using SCM or contraception for preventing horizontal transmission. However, the context of the one seroconversion case does highlight the value of SCC even in the context of ART use. This case was within the SCC2 group and in a couple that successfully achieved a desired pregnancy, but that did not attend any counseling sessions after the initial consult and reported no SCM use. The seroconversion took place despite the index participant being on long-term ART and virally suppressed just months prior to baseline. While the added benefit of SCC for prevention of horizontal HIV transmission may be minimal in the context of ART, this case reveals that some couples remain vulnerable to transmission through potential missed ART doses and blips in viremia. If this couple had attended more SCC sessions and used SCM, the viral transmission may have been prevented.

The ability of SCC to facilitate knowledge of the woman’s most fertile period and being able to target conception behavior to her most fertile days would be expected to bolster the couple’s chances of achieving a pregnancy. Nevertheless, our data did not reveal such an advantage for the intervention participants nor those using SCM. Regardless of arm, about 30% of clients trying to conceive achieved pregnancy, which is consistent with the 27% pregnancy rate found in a South African study of couples receiving SCC [13], but lower than the 43% rate observed in our prior Ugandan observational study of HIV affected couples [31]. Indicators of potential infertility (present in as much as half of our sample) were associated with failure to conceive. Both HIV infection and use of ART have been shown to impede fertility [32, 33], although the degree to which is unclear, as is the rate of infertility among PLHIV.

There are several limitations to the study worth noting. No survey data were collected from the partner of the index participant, except for female partners of male participants who were briefly interviewed on contraception use. Like other studies of SCC [13], we relied on self-report data to measure use of SCM and contraceptives, which render the data susceptible to social desirability bias; however, we took several steps to mitigate the potential for this bias including the following: using research staff who were independent from the clinic to administer surveys, reminding participants that their responses would not be shared with clinic staff, and asking participants to describe in their own words the methods they were using to prevent transmission during attempts to conceive—this was done before they were asked any questions about SCM and the interviewers rated their responses as adherent or not adherent to SCM procedures. It should also be noted that use of SCM was not only reported in the surveys but also during monthly SCC sessions with counselors who often made added calls to the couple to assist in timing of the fertile period and provided equipment (e.g., syringes for MSI) to help the couple implement their selected method. Finally, if socially desirable responding were operating widely among study participants, then it would presumably be operating in all groups, so we would have seen participants in the control arm reporting use of SCM as well and this was not the case.

Other limitations included the lack of blinding in the assessment and analysis of outcomes, which may have biased the interviewer’s rating of accuracy of SCM use, and extent to which group differences were investigated. Also, the sample was comprised solely of PLHIV receiving HIV care in an NGO setting. Our findings may not reflect PLHIV who are not in HIV care and perhaps less likely to be familiar with safer conception and contraception methods and how to use them. HIV care in a public health facility may also differ from NGO settings such as TASO in ways that could impact the reproductive health services and outcomes, such as the level of resources and personnel. However, the fact that the usual care arm reported almost no SCM use suggests minimal influence of potential advantages of NGO settings. Furthermore, evaluating the intervention in serodiscordant couples not using ART, with no infertility issues, could reveal dramatically increased benefits of SCM use for prevention of horizontal transmission and successful safe conception.

## Conclusions

The results of this novel study provide evidence that the *Our Choice* intervention benefits uptake of accurate SCM use among HIV serodiscordant couples. The efficacy and cost-effectiveness of the high intensity implementation approach highlight the critical need for adequate training and ongoing supervision to successfully implement SCC and promote accurate use of complex health behaviors. The findings favoring the higher intensity implementation approach for these complex behaviors may be applicable for other chronic diseases where adoption of complex behavioral skills is critical for clients’ health. The intervention did not affect contraception use among those trying to avoid pregnancy, suggesting the need for supplementary efforts to impact this target behavior. A minority of couples trying to conceive were successful, including those accurately using SCM, highlighting the potential role of infertility in this population and the need for further research in this area.

## Supplementary Information

**Additional file 1: Supplemental Figure 1.** Flow of study participation

**Additional file 2: Supplemental Table 1.** *Our Choice* safer conception counseling topics by session

**Additional file 3: Supplemental Table 2.** Target skills, training, supervision and fidelity checks for counselors and family planning nurses, by intervention arm

**Additional file 4: Supplemental Table 3.** Criteria for determining accurate use of timed condomless intercourse (TCI) and manual self-insemination (MSI)

**Additional file 5: Supplemental Table 4.** Bivariate correlates of pregnancy among study completers who tried to conceive during study (*n*=265)

## Data Availability

An anonymous dataset is available to researchers upon request to the corresponding author and review by the study team.

## Abbreviations

ART: Antiretroviral therapy
eIMB: Ecological adaptation of the Information, Motivation and Behavioral skills
FET: Fisher’s exact test
FP: Family planning
ICC: Intra-class correlation
ITT: Intent-to-treat
MoH: Ministry of Health
MSI: Manual self-insemination
PLHIV: Persons living with HIV
PrEP: Pre-exposure prophylaxis
SCC: Safer conception counseling
SCM: Safer conception methods
TASO: The AIDS Support Organization
TCI: Timed condomless intercourse
