# Supplementary material for: “Our Choice” improves use of safer conception methods among HIV serodiscordant couples in Uganda: a cluster randomized controlled trial evaluating two implementation approaches
Source: Implement Sci. 2021 Apr 15;16:41. doi: 10.1186/s13012-021-01109-z (PMC8048255; doi:10.1186/s13012-021-01109-z)
Supplement: Supplementary file 1 — Additional file 1: Supplemental Figure 1. Flow of study participation [file 13012_2021_1109_MOESM1_ESM.pptx]

## Slide 1
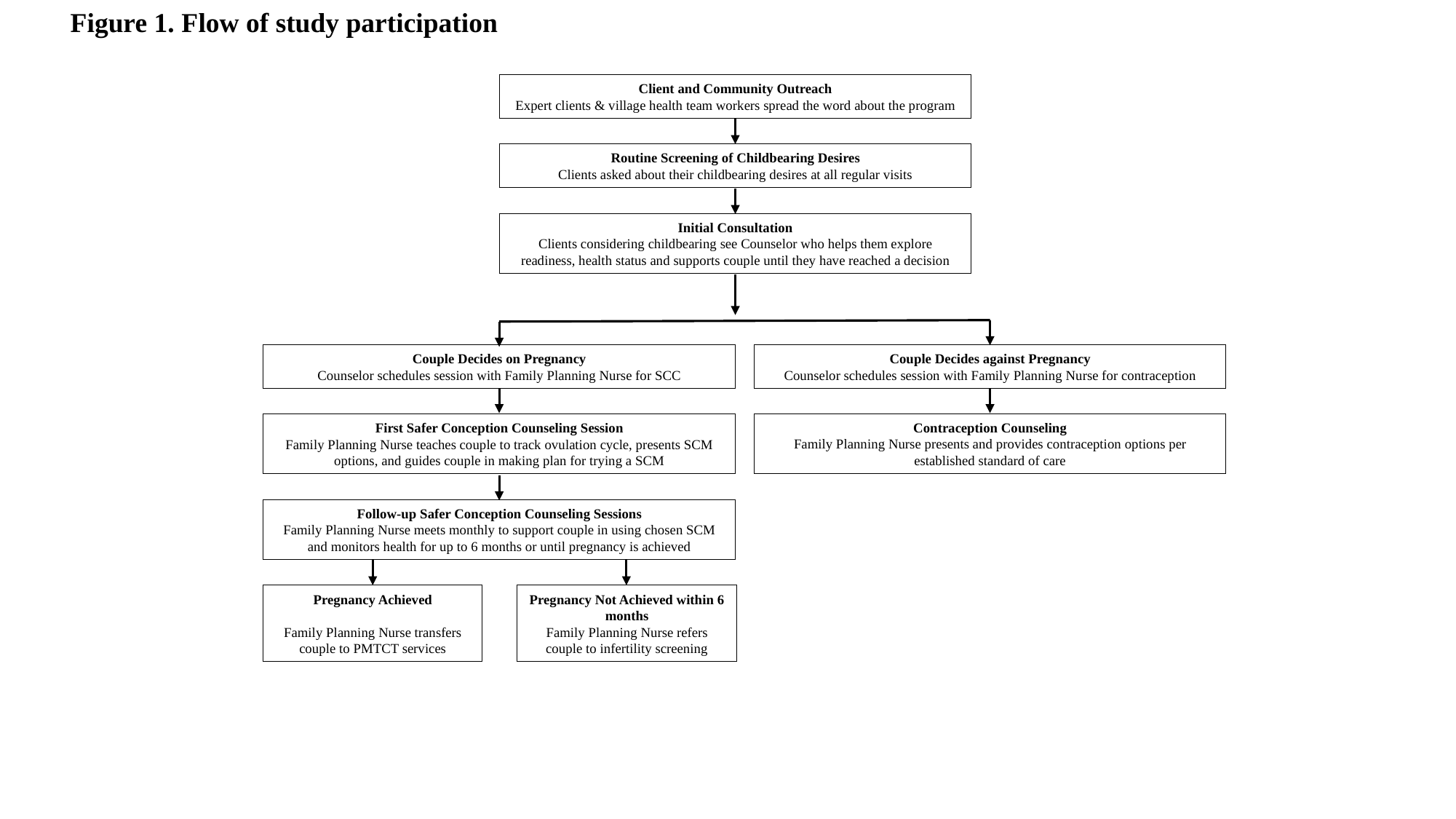

Figure 1. Flow of study participation
Client and Community Outreach
Expert clients & village health team workers spread the word about the program
Routine Screening of Childbearing Desires
Clients asked about their childbearing desires at all regular visits
Initial Consultation
Clients considering childbearing see Counselor who helps them explore readiness, health status and supports couple until they have reached a decision
Couple Decides on Pregnancy
Counselor schedules session with Family Planning Nurse for SCC
Couple Decides against Pregnancy
Counselor schedules session with Family Planning Nurse for contraception
Contraception Counseling
Family Planning Nurse presents and provides contraception options per established standard of care
First Safer Conception Counseling Session
Family Planning Nurse teaches couple to track ovulation cycle, presents SCM options, and guides couple in making plan for trying a SCM
Follow-up Safer Conception Counseling Sessions
Family Planning Nurse meets monthly to support couple in using chosen SCM and monitors health for up to 6 months or until pregnancy is achieved
Pregnancy Achieved
Family Planning Nurse transfers couple to PMTCT services
Pregnancy Not Achieved within 6 months
Family Planning Nurse refers couple to infertility screening
