# Supplementary material for: “Our Choice” improves use of safer conception methods among HIV serodiscordant couples in Uganda: a cluster randomized controlled trial evaluating two implementation approaches
Source: Implement Sci. 2021 Apr 15;16:41. doi: 10.1186/s13012-021-01109-z (PMC8048255; doi:10.1186/s13012-021-01109-z)
Supplement: Supplementary file 2 — Additional file 2: Supplemental Table 1. Our Choice safer conception counseling topics by session [file 13012_2021_1109_MOESM2_ESM.docx]

**Table 1. *Our Choice* safer conception counseling topics by session**

| **Initial Consultation** (45-60 min) | * Build rapport, explain services, communicate non-judgmental support for couple’s decisions  * Explore contextual issues (i.e., client’s childbearing interest, partner’s childbearing interest, partner’s HIV status, family support, disclosure, existing children, health of relationship, available resources, planned separations due to work).  * Review HIV and health risks of childbearing for mother/infant/partner and factors impacting risk (i.e., health, SCM, ART, CD4 cell count, PMTCT, STIs, alcohol use, nutrition). Encourage delaying pregnancy if medical condition not optimal (e.g., not on ART > 6 months, CD4<200, active STI) and provide treatment (for STIs or ART) as needed.  * Introduce safer conception methods.  * Encourage couple to take time to decide and return for SCC or contraception. |
| --- | --- |
| **SCC Session 1**  (20-30 min) | * Review couple’s fertility decision. Provide contraception if no longer desire a child.  * Teach couple to track woman’s ovulation cycle using educational tools.  * Present SCM using educational tools and assist couple to select their best method. Share videos, offer tools, MSI kit, offer text messages to remind client of start of fertile period.  * Discuss other risk reduction options (i.e., circumcision, sperm washing, and PrEP).  * Develop action plan with couple. |
| **Follow-up Sessions**  (20 min) | * Review couple’s successes and challenges with action plan using Problem Solving worksheet.  * Assess usefulness of tools, text messages and identify strategies to overcome barriers.  * Assess HIV-positive’s partner’s ART adherence and refer for adherence counseling if needed.  * Adjust action plan as needed; assess for STIs and treat as needed.  * If partner isn’t attending sessions or hindering use of SCM, discuss strategies for addressing.  * If woman’s period is late, conduct pregnancy test. If pregnant, conduct HIV testing with partner and start HIV-positive mothers on PMTCT.  * After 6 months of correct SCM use, if pregnancy has not been achieved, discontinued SCC and refer couple to infertility clinic. |

This table is from this prior publication that described the Our Choice study protocol and intervention: Goggin K, Hurley EA, Beyeza-Kashesya J, Gwokyalya V, Finocchario-Kessler S, Birungi J, et al. Study protocol of "Our Choice": a randomized controlled trial of the integration of safer conception counseling to transform HIV family planning services in Uganda. Implement Sci. 2018;13(1):110.
