# Supplementary material for: “Our Choice” improves use of safer conception methods among HIV serodiscordant couples in Uganda: a cluster randomized controlled trial evaluating two implementation approaches
Source: Implement Sci. 2021 Apr 15;16:41. doi: 10.1186/s13012-021-01109-z (PMC8048255; doi:10.1186/s13012-021-01109-z)
Supplement: Supplementary file 3 — Additional file 3: Supplemental Table 2. Target skills, training, supervision and fidelity checks for counselors and family planning nurses, by intervention arm [file 13012_2021_1109_MOESM3_ESM.docx]

**Table 2. Target skills, training, supervision and fidelity checks for counselors and family planning nurses, by intervention arm**

|  | **Skills** | **Training Strategy** | **Hours** | **Supervision & Fidelity** |
| --- | --- | --- | --- | --- |
| **SCC1:**  Study team-led implementation model | - Building rapport - Exploring readiness   - Using open question   - Using reflective listening   - Offering information - Assessing health factors - Assisting w/   - disclosure   - family planning decision - Confidence providing SCC - Teaching SCM and use of tools - Problem solving strategies - Using text message system | - Exploration of counselors’ beliefs and attitudes - Didactic review of manual - Videos / live demos - Role plays   - Communication skills   - Intervention sessions   - Teaching use of tools - Personalized feedback: MI skills & content mastery - Identification of implementation barriers and solutions - Hands-on text training | 16 | - Supervision   - Semimonthly individual and group;   - Role-plays - Sessions recorded for coding & tailored feedback - Session checklists - Counselor completes - Supervisor provides tailored feedback on coverage of content and MI skills - 95% of text messages sent on time |
| **SCC2:**  MoH-led implementation model | - Knowledge of SCM - Confidence providing SCC - Familiar w/ manual and tools | - Lecture on benefit of SCC and history of stigma - Read manual sections Answer provider questions - Videos / live demos - Practice tools | 8 | - Supervision on request - Quarterly MoH support visits - Yearly training update |
