# Supplementary material for: “Our Choice” improves use of safer conception methods among HIV serodiscordant couples in Uganda: a cluster randomized controlled trial evaluating two implementation approaches
Source: Implement Sci. 2021 Apr 15;16:41. doi: 10.1186/s13012-021-01109-z (PMC8048255; doi:10.1186/s13012-021-01109-z)
Supplement: Supplementary file 4 — Additional file 4: Supplemental Table 3. Criteria for determining accurate use of timed condomless intercourse (TCI) and manual self-insemination (MSI) [file 13012_2021_1109_MOESM4_ESM.docx]

**Table 3. Criteria for determining accurate use of timed condomless intercourse (TCI) and manual self-insemination (MSI)**

Timed Condomless Intercourse

1. Knows how to accurately determine the timing of the most fertile days (e.g., if 28 day cycle, and ovulation starts at day 14, than most fertile period is from day 13 to 15).^1^

2. Knows that most fertile period is 3 days long.

3. Reports having unprotected intercourse during the 3 most fertile days.

4. Reports always using condoms during sex that takes place outside the 3 most fertile days.

Manual Self-Insemination

1. Knows how to accurately determine the timing of the most fertile days (e.g., if 28 day cycle, and ovulation starts at day 14, than most fertile period is from day 13 to 15).^1^

2. Knows that most fertile period is 3 days long.

3. Man collected semen using condom during sex or a cup/container.

4. Syringe was used to inject semen into vagina.

5. Injected semen within one hour after ejaculation.

6. Semen was injected into vagina during the 3 most fertile days.

7. Woman was in proper position (on back with hips raised).

8. Woman remained in proper position for at least 30 minutes after injection of semen.

9. Reports always using condoms during sexual intercourse.

^1^ Per the *Our Choice* protocol, SCC1/SCC2 nurses were instructed to text/call the client the day before the woman’s most fertile period to ensure that the couple knew when to starting using their chosen SCM. Accuracy ratings for clients who received these calls did not include item number 1.
