# Supplementary material for: “Our Choice” improves use of safer conception methods among HIV serodiscordant couples in Uganda: a cluster randomized controlled trial evaluating two implementation approaches
Source: Implement Sci. 2021 Apr 15;16:41. doi: 10.1186/s13012-021-01109-z (PMC8048255; doi:10.1186/s13012-021-01109-z)
Supplement: Supplementary file 5 — Additional file 5: Supplemental Table 4. Bivariate correlates of pregnancy among study completers who tried to conceive during study (n=265) [file 13012_2021_1109_MOESM5_ESM.docx]

**Table 4. Bivariate correlates of pregnancy among study completers who tried to conceive during study (n=265)**

| **Variable** | **Did not become pregnant (n=187)** | **Became pregnant (n=87)** | P value^1^ |
| --- | --- | --- | --- |
|  | Mean (SD)/% | Mean (SD)/% |  |
| HIV+ member of couple is female | 53**·**9% | 46**·**0% | **·**224 |
| Age of female partner | 32**·**9 (6**·**3) | 30**·**0 (6**·**2) | **·**001 |
| Client/couple used TCI/MSI during study | 50**·**0% | 43**·**7% | **·**333 |
| Client/couple used TCI/MSI accurately during study | 16**·**9% | 17**·**2% | **·**937 |
| Both members wanted a child at baseline | 89**·**9% | 92**·**0% | **·**589 |
| Female partner was using modern contraceptives at baseline | 10**·**1% | 17**·**2% | **·**099 |
| Couple had been trying to get pregnant for 6+ months prior to baseline | 17**·**1% | 12**·**7% | **·**376 |
| Couple had previously tested for infertility or told they may be infertile by doctor | 5**·**6% | 0 | **·**032^1^ |
| Either partner was diagnosed with STI in 6 months prior to baseline | 12**·**5% | 16.0% | **·**735^1^ |

^1^ P values are from chi square test, 2-tailed independent t-test, or Fisher’s Exact Test
